# Effects of Hydrogel-Forming Composite Cryoprotectants on the Quality of Frozen Freshwater Crayfish (*Procambarus clarkii*)

**DOI:** 10.3390/gels12090836

**Published:** 2026-09-12

**Authors:** Diejun Jia, Haoran Zhuo, Yuxi Sun, Longwei Jiang, Lijuan Yang, Junhao Yang, Yuan Fu, Jiacheng Li, Lijie Shen, Ke Zhao, Yuxia Zhu, Shuibing Yang, Tao Huang

**Affiliations:** 1 School of Life Sciences and Medical Engineering, Anhui University, Hefei 230601, China; diejun2242885917@163.com; 2 School of Modern Agriculture and Food Engineering, Chuzhou University, Chuzhou 239000, China; hrzhuo267@163.com (H.Z.); blackmyth@foxmail.com (Y.S.); m15212037305@163.com (L.Y.); 19719585440@163.com (J.Y.); fuyuan@chzu.edu.cn (Y.F.); m18756719932@163.com (J.L.); 15056109420@163.com (L.S.); nuaazhaok@126.com (K.Z.); yxzhu@chzu.edu.cn (Y.Z.); 3 Key Laboratory of Jianghuai Agricultural Product Fine Processing and Resource Utilization of Ministry of Agriculture and Rural Affairs, Anhui Engineering Research Center for High Value Utilization of Characteristic Agricultural Products, School of Food and Nutrition, Anhui Agricultural University, Hefei 230036, China; jianglw58@163.com; 4 College of Food Science and Engineering, Ningbo University, Ningbo 315800, China; huangtao@nbu.edu.cn

**Keywords:** *Procambarus clarkii*, cryoprotectant, hydrogel, TBARS, TVB-N, response surface methodology (RSM)

## Abstract

The use of a composite cryoprotectant to maintain the quality of freshwater crayfish (*Procambarus clarkii*) during frozen storage was investigated in this study. Chitosan (CTS) and carrageenan oligosaccharides (CG) are natural hydrogel-forming polysaccharides that serve as moisture barriers. The effects of different concentrations of CTS (0–1.5%), CG (0–1.0%), antifreeze proteins (AFPs, 0–0.25%), and tea polyphenols (TP, 0–0.03%) on crayfish muscle tissue were evaluated by measuring water-holding capacity (WHC), total volatile basic nitrogen (TVB-N), and thiobarbituric acid reactive substances (TBARS). Based on single-factor experiments, a three-factor, three-level response surface methodology (RSM) was applied with WHC as the response variable. The results showed that a maximum WHC of 71.65 ± 0.46% was obtained under the optimal treatment, comprising 0.81% CG, 0.61% CTS, and 0.17% AFPs. This study provides a theoretical basis for developing composite cryoprotectants and frozen pre-cooled products from freshwater crayfish, as well as a reference for the application of polysaccharide-based hydrogels in aquatic product frozen preservation.

## 1. Introduction

Rising living standards and improved dietary habits have led to steadily growing consumer demand for aquatic products, driven by their recognized health benefits [1,2]. The freshwater crayfish (*Procambarus clarkii*), a key aquatic resource in China, is particularly popular for its distinctive flavor, high protein content, low fat, and abundance of trace elements [3]. However, its high moisture and protein content also render it highly perishable, susceptible to rapid microbial spoilage and quality deterioration post-harvest [4]. While conventional freezing is a primary method for extending shelf life, it inevitably induces quality degradation. The formation and growth of ice crystals during freezing can cause mechanical damage to muscle tissue, promote protein denaturation and aggregation, and lead to irreversible loss of water-holding capacity, texture, and juiciness upon thawing [5,6].

To mitigate these adverse effects, the use of cryoprotectants has become a common strategy. Natural cryoprotectants, especially polysaccharide-based ones, are gaining attention due to their safety, biocompatibility, and biodegradability. Chitosan (CTS) and carrageenan oligosaccharides (CG) are well-documented gel-forming biopolymers. Chitosan can bind free water through hydrogen bonding and non-covalent interactions, thereby enhancing cryoprotective effects and inhibiting ice crystal formation [7]. In dilute immersion systems for aquatic products, their protective functions are mainly governed by intermolecular non-covalent interactions. Previous frozen shrimp soaking experiments demonstrated that carrageenan oligosaccharides could bind muscle proteins and trap water molecules through hydrogen bonds, restrain ice crystal recrystallization and alleviate myofibrillar structural destruction under temperature fluctuation conditions [8]. As another representative polysaccharide, chitosan provides intrinsic antibacterial capacity to suppress microbial reproduction during cold storage [9]. In addition, abundant studies have confirmed that chitosan can form hydrogen bonding, electrostatic and hydrophobic non-covalent interactions with tea polyphenols, which facilitates polyphenol immobilization and improves the stability of antioxidant components in aqueous systems [10]. Apart from polysaccharide cryoprotectants, protein-derived antifreeze peptides exert favorable cryoprotective performance: they interact with ice crystal interfaces and muscle proteins through non-covalent forces, delay protein oxidative denaturation, and mitigate mechanical injury caused by enlarged ice crystals in frozen shrimp muscle [11]. These published findings provide theoretical support for selecting polysaccharides, protein-based components and polyphenols as composite cryoprotective agents.

These polysaccharide-derived biopolymers can exert protective effects by encapsulating free water through intermolecular association to restrict moisture migration and mitigate ice-crystal-induced myofibrillar protein denaturation [12]. Specifically, carrageenan oligosaccharides have been shown to exhibit cryoprotective effects on myofibrillar proteins in aquatic products [13].

Despite their individual benefits, single-component cryoprotectants often face limitations in providing comprehensive protection. A single polysaccharide component provides only partial protection, failing to simultaneously mitigate ice crystal-induced damage, protein oxidation, and microbial growth. For instance, the efficacy of natural antioxidants like tea polyphenols in frozen systems can be constrained by their solubility, stability, and localized distribution [14]. Similarly, although antifreeze proteins (AFPs) can inhibit ice crystal growth by adsorbing to ice surfaces [15], the protection they offer is compromised when free AFPs undergo self-aggregation and deactivation without confinement within the polysaccharide matrix. This underscores the potential advantage of composite cryoprotectant systems designed to leverage complementary mechanisms [16,17]. Recent research trends indicate a move towards combining functional ingredients to achieve multifunctional protection. For example, studies have explored composites of chitosan with antioxidants or proteins for preserving various seafood [16,18]. However, few studies have systematically developed and optimized a blended composite formulation using CG and CTS as the primary protective components, supplemented with AFPs to mitigate ice-induced damage and tea polyphenols as auxiliary antioxidants for the frozen preservation of *Procambarus clarkii*. Related research on this gel-based preservation strategy remains scarce for crayfish products.

The combined effects of CTS, CG, AFPs, and TP are enhanced via polysaccharide-mediated intermolecular associations, thereby achieving comprehensive cryoprotection against quality deterioration of frozen crayfish. Therefore, the present study systematically investigated the cryoprotective effects of carrageenan oligosaccharides (CG), chitosan (CTS), antifreeze proteins (AFPs), and tea polyphenols (TP) on frozen freshwater crayfish (*Procambarus clarkii*) muscle, and evaluated their efficacy using established quality indicators (WHC, TVB-N, TBARS) that serve as proxies for freezing damage mitigation. A blank control (0% cryoprotectant) was included in all experiments. In addition, the optimal formulation was obtained using response surface methodology (RSM), aiming to develop a safe and efficient frozen preservation strategy for freshwater crayfish.

## 2. Results and Discussion

### 2.1. Effect of Chitosan on the Quality of Frozen Freshwater Crayfish

Chitosan (CTS), a natural polysaccharide derived from chitin, extends the shelf life of foods through the formation of edible films or coatings. Chitosan-based matrices possess moisture resistance and gas barrier properties, inhibiting oxygen transmission and microbial growth [19]. The three-dimensional network structure of chitosan hydrogel may enhance cryoprotective effects by trapping free water and reducing ice crystal formation [7]. However, it is important to note that in practical frozen food applications, chitosan rarely functions as an isolated agent; rather, it serves as a foundational component within composite cryoprotectant systems designed to leverage complementary mechanisms [12].

**Figure 1:**
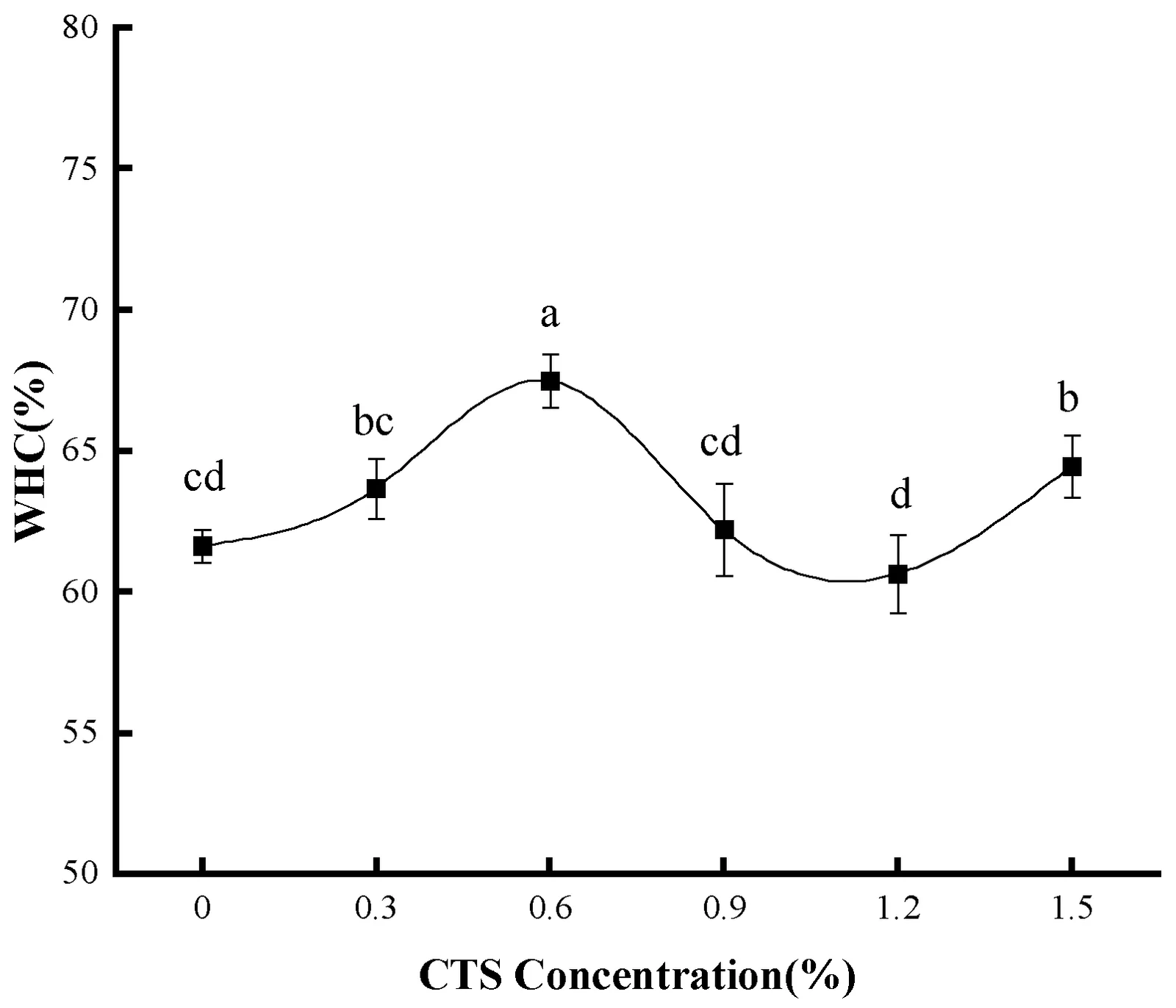
Effect of CTS Concentration on WHC of Freshwater Crayfish. Note: Data are presented as the mean ± SD (n = 3). CTS refers to chitosan; WHC refers to water-holding capacity. The 0% group corresponds to the untreated blank control (no cryoprotectant additives). Different lowercase letters above the bars indicate statistically significant differences (*p* < 0.05).

As shown in Figure 1, the maximum WHC of 67.5 ± 1.0% was achieved at 0.6% chitosan, which was significantly higher (*p* < 0.05) than those at other concentrations, and also significantly higher than the blank control group. At 0.3% CTS, the WHC was 63.7 ± 1.2%, which was significantly higher than that of the blank control but notably lower than the optimal 0.6% group. This suggests that the low concentration of chitosan forms an insufficient protective layer on the muscle surface, failing to provide complete coverage and adequate barrier function against ice crystal growth and moisture migration during freezing [20]. As a result, the protective effect was only partial, leading to greater freeze-induced water loss compared to the 0.6% group. However, the WHC of this group remained significantly higher than that of the blank control. Conversely, concentrations above 0.6% resulted in decreased WHC, which can be attributed to osmotic pressure effects. According to Lan et al. [21], chitosan polymeric networks exhibit significant deswelling behavior in response to osmotic pressure gradients; as polymer concentration increases, the denser network structure creates higher external osmotic pressure, promoting water efflux from muscle cells to the coating solution. This is consistent with findings by García et al. [20], who observed that chitosan coatings can influence water loss dynamics during osmotic processes by altering the osmotic balance at the tissue interface. Additionally, excessively thick or uneven coatings formed at high concentrations may impair binding to muscle proteins and accelerate water evaporation [21]. At the optimal concentration of 0.6%, chitosan likely achieves a balance between film-forming capacity and interfacial compatibility with muscle tissue. The observed improvements in WHC and oxidation indicators suggest that this concentration may form a protective layer with optimal barrier properties against oxygen and water vapor transmission. This concentration-dependent optimization is critical when designing composite cryoprotectant systems, as the interaction between chitosan and other functional ingredients (e.g., polyphenols, polysaccharides) is highly concentration-sensitive [9].

**Figure 2:**
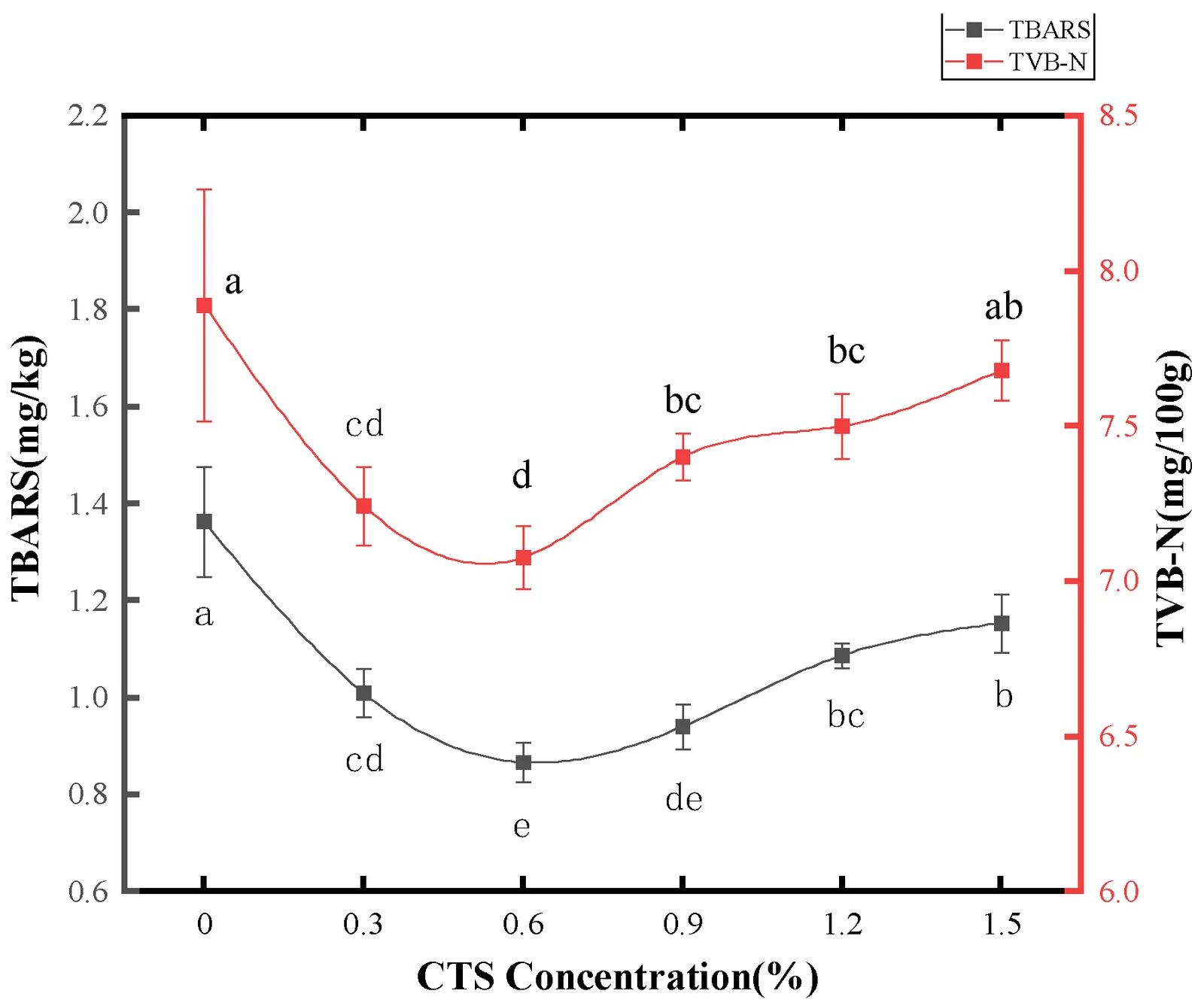
Effect of CTS concentration on TVB-N and TBARS of freshwater crayfish. Note: Data are presented as the mean ± SD (n = 3). CTS refers to chitosan; TVB-N refers to total volatile basic nitrogen; TBARS refers to thiobarbituric acid-reactive substances. The 0% group corresponds to the untreated blank control (no cryoprotectant additives). Different lowercase letters above the bars indicate statistically significant differences (*p* < 0.05).

Figure 2 shows that TVB-N and TBARS values exhibited similar concentration-dependent trends, with the blank control showing the highest levels among all test groups, confirming that untreated crayfish suffered from severe protein decomposition and lipid oxidation during frozen storage. Both indicators decreased progressively from 0% to 0.6% CTS, reaching minimum values of 7.08 ± 0.10 mg/100 g and 0.87 ± 0.04 mg/kg at 0.6% chitosan, respectively (*p* < 0.05). These results indicate that 0.6% chitosan effectively inhibits protein degradation and lipid oxidation. The polymeric network formed by chitosan acts as a physical barrier that blocks oxygen penetration. However, the observed increase in oxidation markers at higher concentrations suggests that the pro-dehydration effects of high osmotic pressure may outweigh the benefits of increased coating thickness, revealing the adverse impacts induced by excessive chitosan dosage, even within composite mixtures. Notably, although both TVB-N and TBARS increased at concentrations above 0.6%, all chitosan-treated groups still exhibited lower values than the blank control, indicating that even at suboptimal concentrations, chitosan provided measurable protection against oxidation and spoilage.

These concentration-related adverse responses under fixed-formula conditions underscore why recent research has moved toward combining chitosan with other functional ingredients to achieve multifunctional protection. For example, Qiu et al. [9] demonstrated that combining chitosan with lysozyme and carrageenan oligosaccharides significantly improved crayfish preservation compared to single-component treatments. Similarly, Ludtke et al. [16] reported that chitosan–fish oil–green tea extract coatings outperformed simple formulations for Atlantic bonito preservation, illustrating that complementary chitosan-based multi-component systems can achieve enhanced protection through complementary mechanisms.

### 2.2. Effect of Carrageenan Oligosaccharides on the Quality of Frozen Freshwater Crayfish

Carrageenan oligosaccharides, a natural polysaccharide from red algae, are widely used in the food industry as thickeners, stabilizers, and gelling agents [22]. While CG demonstrate independent cryoprotective effects through ice crystal inhibition and protein stabilization [23], in practical frozen seafood preservation, CG rarely act as a standalone agent. Rather, it functions as a critical component within composite matrices, often combined with chitosan, proteins, or other polysaccharides to create complementary cryoprotective effects [24,25].

**Figure 3:**
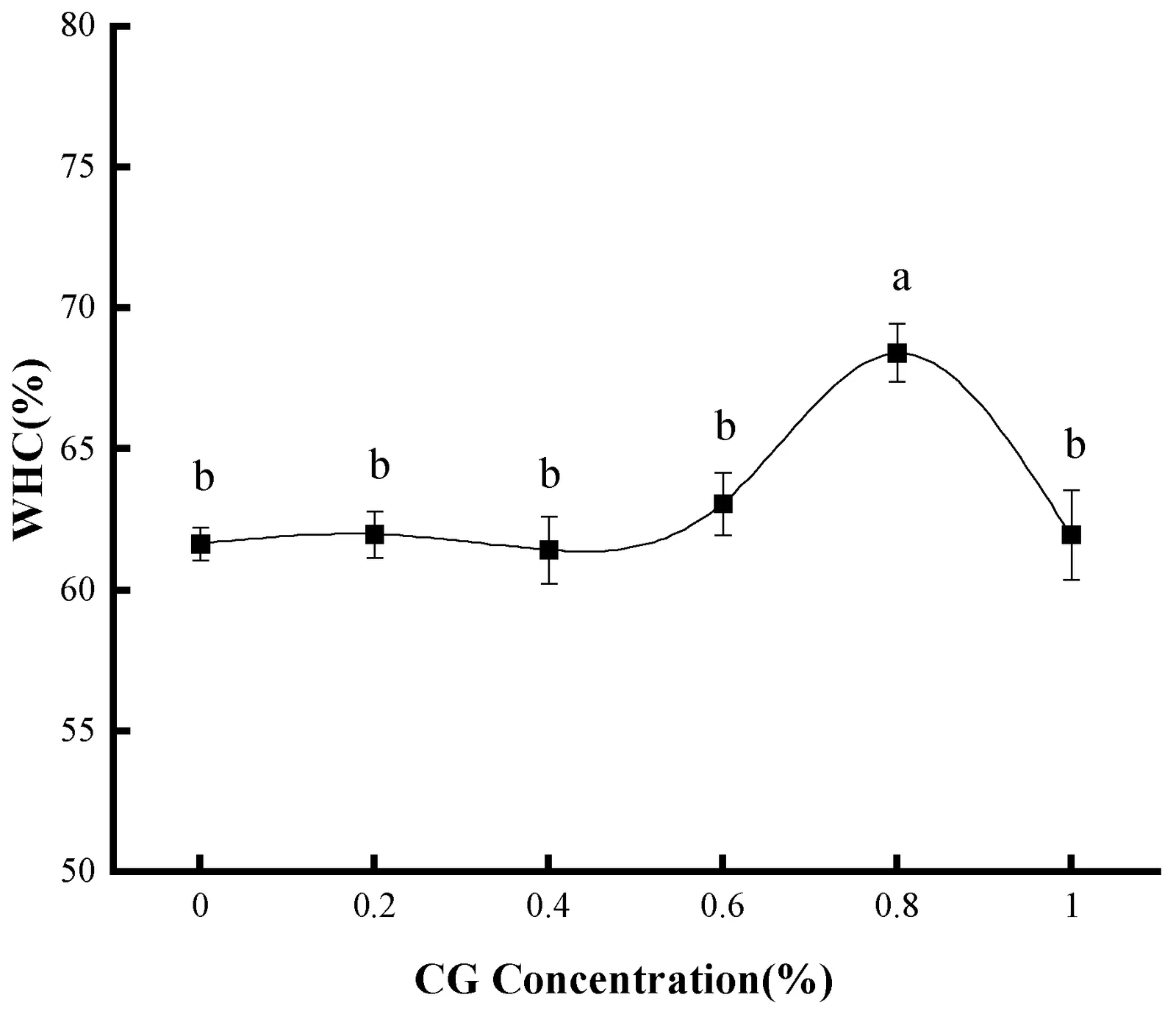
Effect of CG concentration on WHC of freshwater crayfish. Note: Data are presented as the mean ± SD (n = 3). CG refers to carrageenan oligosaccharides; WHC refers to water-holding capacity. The 0% group corresponds to the untreated blank control (no cryoprotectant additives). Different lowercase letters above the bars indicate statistically significant differences (*p* < 0.05).

As shown in Figure 3, the blank control exhibited a WHC of 61.63 ± 0.59%. Low concentrations of CG (0.2%, 0.4%, and 0.6%) showed only marginal improvements over the blank, suggesting that inadequate intermolecular association at these concentrations failed to provide adequate protection against freeze-induced dehydration. The WHC of freshwater crayfish exhibited a concentration-dependent biphasic response to CG, with the maximum value of 68.4 ± 1.1% achieved at 0.8% (*p* < 0.05). At 0.4%, the lower WHC (61.63 ± 0.59%) likely results from inadequate intermolecular association, leaving muscle proteins inadequately protected against freeze-induced dehydration, yet this value was still slightly higher than that of the blank group. Conversely, concentrations exceeding 0.8% led to reduced WHC, which may be attributed to excessive co-association effects. According to Zhang et al. [24], while CG interact with myofibrillar proteins to enhance structural stability through hydrogen bonding, excessive CG concentration can lead to compact polymer assemblies that disrupt the protein–water interface and induce osmotic stress. This is consistent with findings by Pérez-Mateos et al. [25], who observed that hydrocolloid type and concentration in fish mince systems can cause polymeric assembly property variations, potentially impairing water retention when network structures become too dense.

At 0.8%, CG likely form polymer molecular assemblies of optimal density that balance water encapsulation with structural flexibility. However, the inability of single-component systems to simultaneously address multiple freezing stresses (ice crystal growth, protein denaturation, lipid oxidation) necessitates the use of composite formulations. For instance, Zhang et al. [24] demonstrated that CG combined with egg white protein significantly improved the gelation properties and freeze–thaw stability of *Culter alburnus* myofibrillar protein compared to CG alone, as the protein component filled network gaps while CG provided ice crystal inhibition.

**Figure 4:**
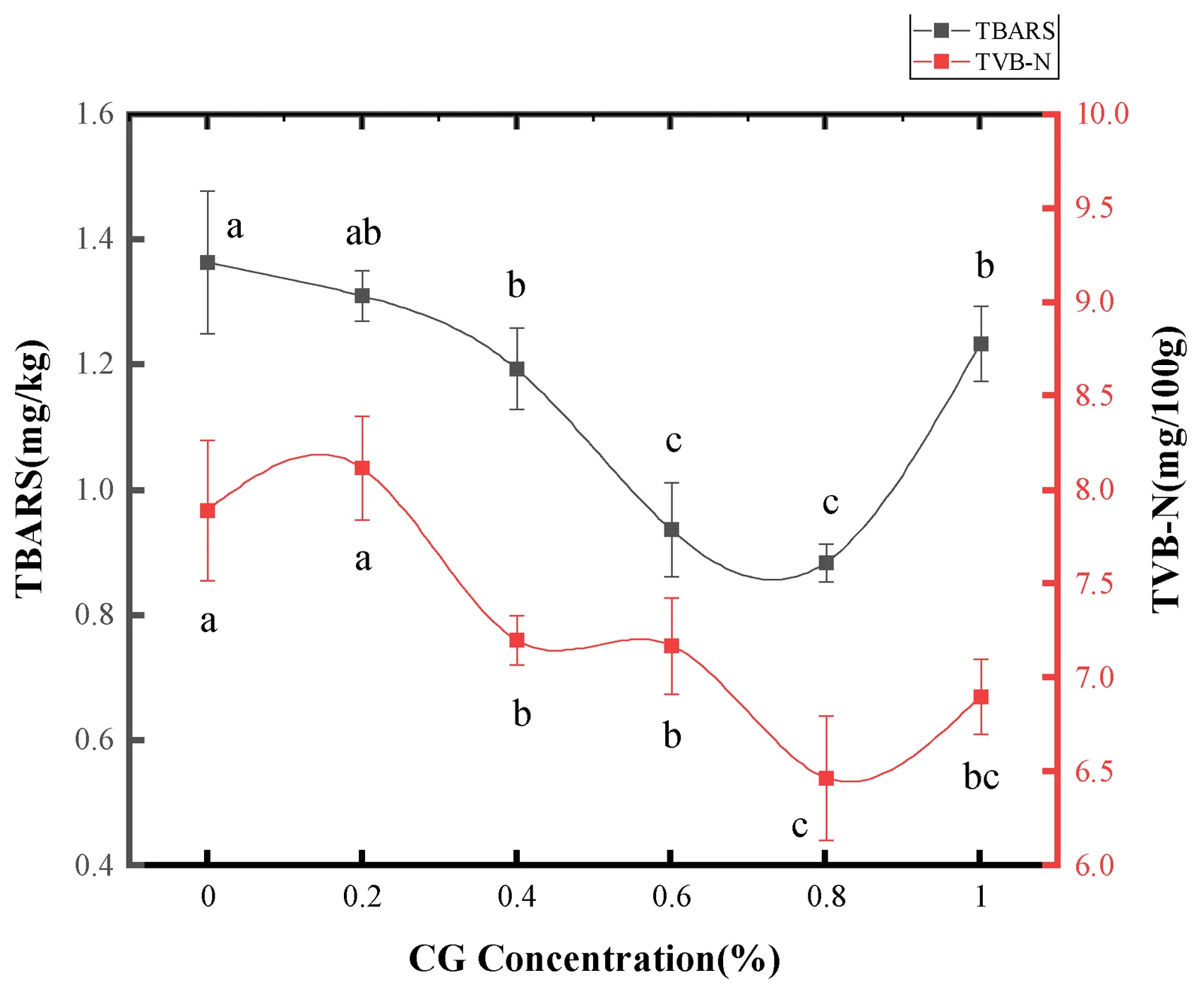
Effect of CG concentration on TVB-N and TBARS of freshwater crayfish. Note: Data are presented as the mean ± SD (n = 3). CG refers to carrageenan oligosaccharides; TVB-N refers to total volatile basic nitrogen; TBARSs refers to thiobarbituric acid reactive substances. The 0% group corresponds to the untreated blank control (no cryoprotectant additives). Different lowercase letters above the bars indicate statistically significant differences (*p* < 0.05).

Figure 4 demonstrates that TVB-N and TBARS values exhibited similar biphasic trends with increasing CG concentration. The blank control showed relatively high initial levels (TVB-N: 7.89 ± 0.37 mg/100 g; TBARS: 1.36 ± 0.11 mg/kg), indicating substantial protein spoilage and lipid oxidation in untreated crayfish during frozen storage. Both indicators decreased progressively in response to CG concentration, and the minimum values (6.47 ± 0.33 mg/100 g and 0.88 ± 0.03 mg/kg, respectively) were achieved at 0.8% (*p* < 0.05). However, at concentrations exceeding 0.8%, both TVB-N and TBARS increased again. The increased oxidation and protein degradation at higher CG concentrations (>0.8%) may result from overly dense barriers that impede metabolite removal, or from network rigidity that compromises the structural integrity of muscle cells during freeze–thaw cycles [24].

Rather than relying on a single hydrocolloid at suboptimal or supraoptimal concentrations, combining CG with complementary agents (e.g., chitosan for antimicrobial barrier properties or trehalose for enhanced water retention) allows for complementary effects that overcome the inherent constraints of individual components. This rationale supports the subsequent optimization of composite cryoprotectant systems using Response Surface Methodology (RSM), where CG function as one component within an integrated protective matrix rather than an isolated additive [24].

### 2.3. Effect of Antifreeze Proteins on the Quality of Frozen Freshwater Crayfish

Antifreeze proteins (AFPs), found naturally in polar organisms, deep-sea fish, and some plants, inhibit ice crystal formation at subzero temperatures by depressing the freezing point and preventing macroscopic ice crystal growth [26,27]. While AFPs demonstrate independent cryoprotective effects through thermal hysteresis activity and ice recrystallization inhibition [27], their practical application in frozen food systems almost invariably involves integration with polymeric matrices (e.g., chitosan, carrageenan oligosaccharides) rather than standalone use [27], as the hydrogel networks can stabilize AFPs’ conformation and prevent self-aggregation [28].

**Figure 5:**
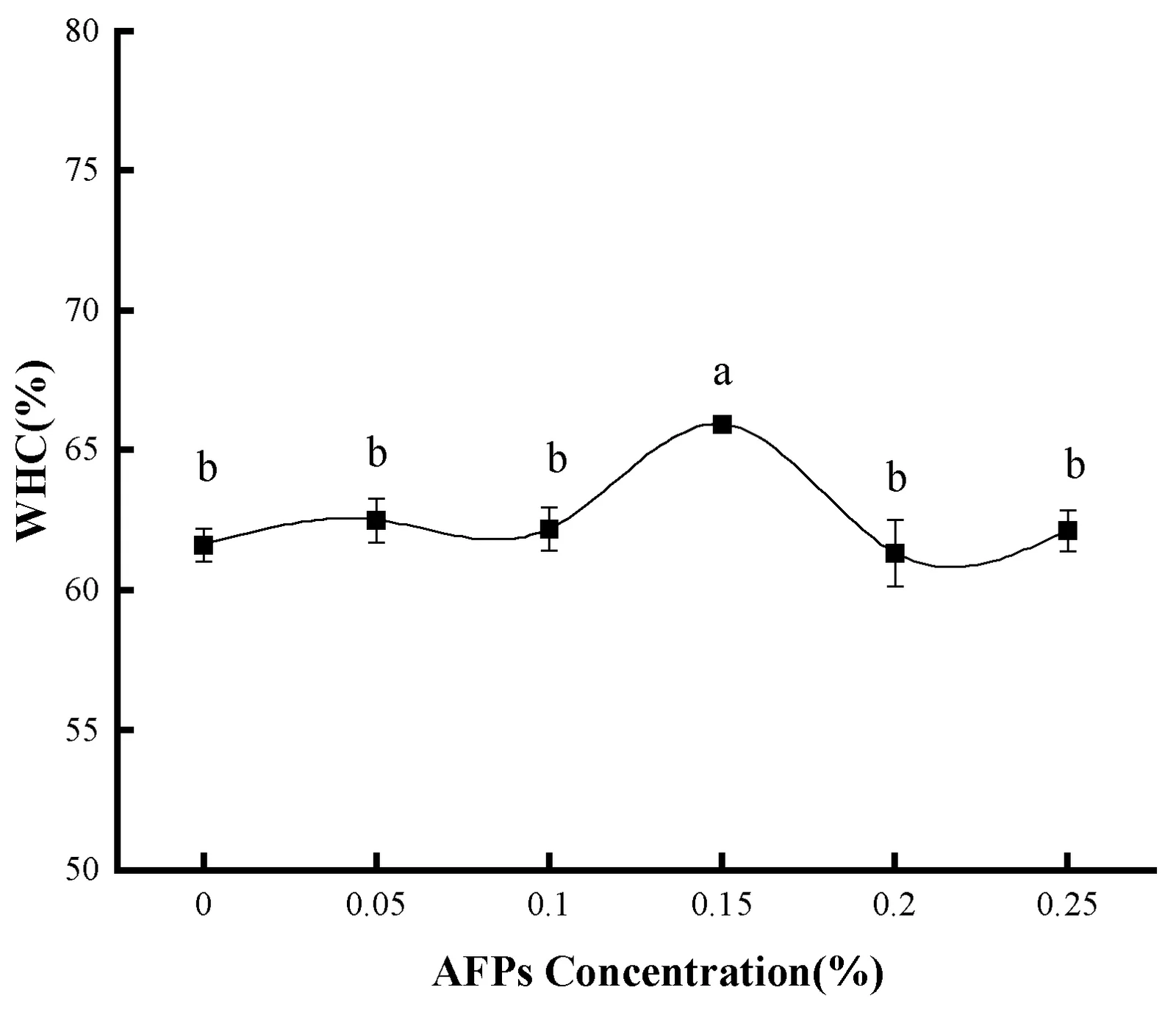
Effect of AFPs concentration on WHC of freshwater crayfish. Note: Data are presented as the mean ± SD (n = 3). AFPs refers to antifreeze proteins; WHC refers to water-holding capacity. The 0% group corresponds to the untreated blank control (no cryoprotectant additives). Different lowercase letters above the bars indicate statistically significant differences (*p* < 0.05).

As shown in Figure 5, the WHC of freshwater crayfish exhibited a concentration-dependent biphasic response to AFPs, initially increasing with rising AFPs concentration, reaching a maximum of 65.9 ± 0.3% at 0.15% AFPs, and then decreasing at higher concentrations (0.20% and 0.25%). The blank control exhibited a WHC of 61.63 ± 0.59%, which was comparable to that of most AFPs-treated groups (0.05%, 0.10%, 0.20%, and 0.25%; *p* > 0.05), indicating that AFPs at these concentrations failed to provide measurable improvement in moisture retention. Lower concentrations (0.05–0.10%) resulted in insufficient cryoprotection, likely due to inadequate AFPs coverage at ice crystal interfaces, leading to greater freeze-induced dehydration. Conversely, concentrations above 0.15% progressively decreased WHC, which can be attributed to protein aggregation phenomena. Du et al. [28] demonstrated that excessive AFPs concentrations promote self-aggregation through hydrophobic interactions, disrupting the normal binding of AFPs to ice crystal surfaces and thereby impairing their ice growth inhibition activity. This aggregation-induced deactivation explains the reduced cryoprotective efficacy observed at AFPs concentrations ≥ 0.20%.

**Figure 6:**
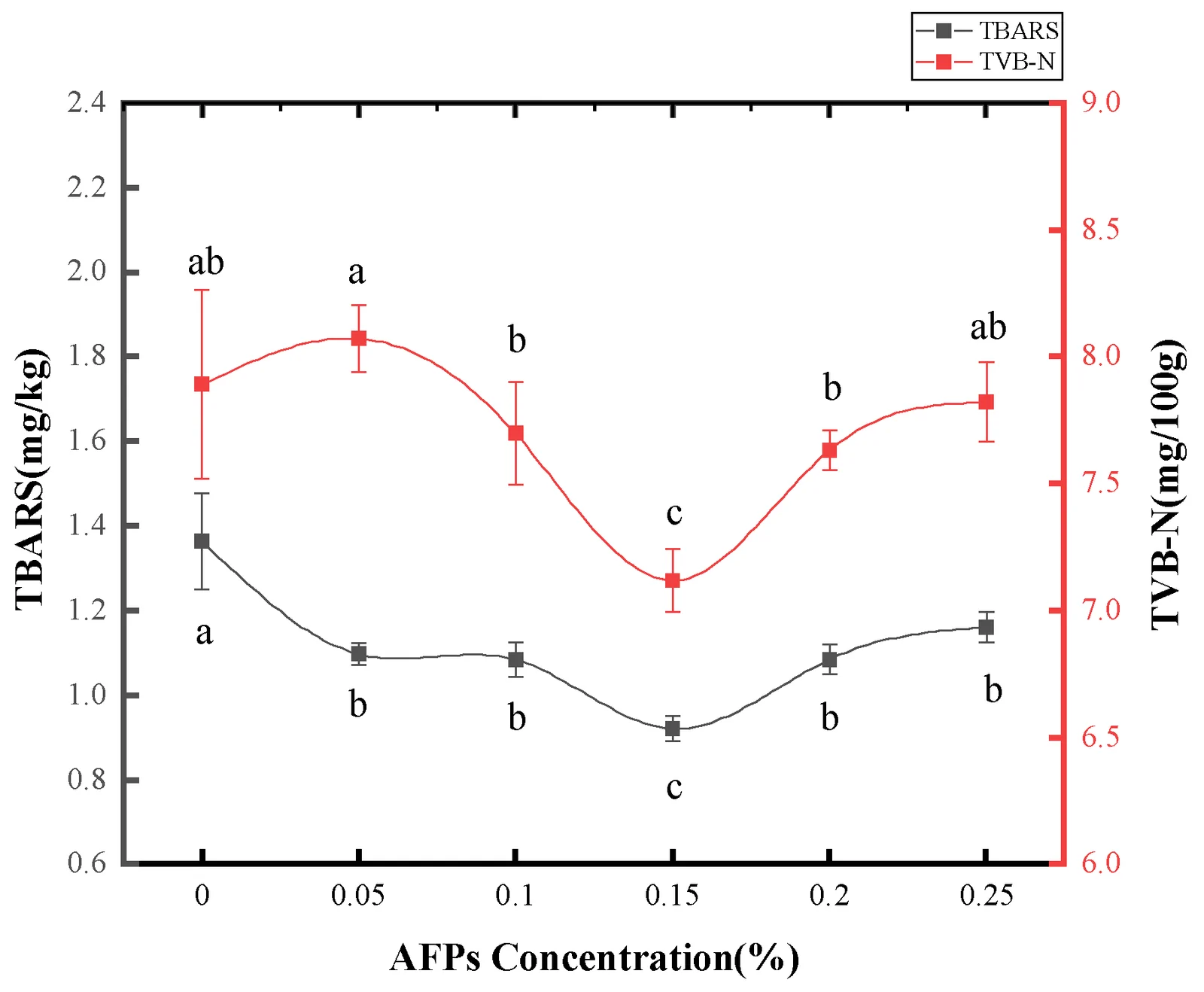
Effect of AFPs concentration on TVB-N and TBARS of freshwater crayfish. Note: Data are presented as the mean ± SD (n = 3). AFPs refers to antifreeze proteins; TVB-N refers to total volatile basic nitrogen; TBARS refers to thiobarbituric acid reactive substances. The 0% group corresponds to the untreated blank control (no cryoprotectant additives). Different lowercase letters above the bars indicate statistically significant differences (*p* < 0.05).

As illustrated in Figure 6, TVB-N and TBARS values exhibited concentration-dependent trends consistent with the WHC response. The blank control showed the highest TBARS level among all groups, indicating severe lipid oxidation in untreated crayfish during frozen storage. TBARS decreased progressively from 0% to 0.15% AFPs, reaching a minimum of 0.92 ± 0.03 mg/kg at 0.15% (*p* < 0.05), then increased again at higher concentrations (0.20% and 0.25%). TVB-N increased from 7.89 mg/100 g in the blank control to a maximum of 8.1 mg/100 g at 0.05% AFPs, then decreased to a minimum of 7.12 ± 0.12 mg/100 g at 0.15% AFPs (*p* < 0.05), followed by a gradual increase at 0.20% and 0.25%. The transient increase at 0.05% may be attributed to insufficient AFPs coverage, where limited AFPs molecules fail to fully suppress ice crystal growth; the resulting ice crystal puncture of cell membranes may facilitate the release and accumulation of intracellular proteinolytic products [27]. As AFPs concentration increased to 0.15%, sufficient coverage at ice crystal interfaces effectively inhibited ice recrystallization, minimized mechanical damage to muscle cells, and thereby reduced the leakage of intracellular substrates that promote microbial growth and enzymatic protein degradation [27].

The reduced oxidation and protein degradation at 0.15% AFPs suggest improved cellular integrity, which is likely associated with AFP-mediated ice crystal inhibition and the mitigation of freeze-induced mechanical damage to muscle tissue [27].

The narrow effective concentration window (0.15%) and the sharp decline in efficacy at higher concentrations due to aggregation (as documented by Du et al. [28]) highlight the challenges even for AFPs within mixed systems when the AFPs dosage is the only variable. When integrated with chitosan and carrageenan oligosaccharides in subsequent RSM optimization, the 0.15% AFPs level can be maintained while leveraging the polysaccharides’ water-binding and barrier properties to create a multi-mechanism protective system that addresses ice crystal growth (AFPs), water retention (CG), and antimicrobial protection (CTS) simultaneously.

### 2.4. Effect of Tea Polyphenol on the Quality of Frozen Freshwater Crayfish

Tea polyphenols (TP), primarily derived from *Camellia sinensis*, are potent natural antioxidants widely recognized for their free radical-scavenging capacity and ability to retard oxidative degradation in food systems [14]. However, while TP demonstrate strong antioxidant activity in vitro, their practical application in frozen seafood preservation is inherently limited when used as an isolated agent. This discrepancy arises from TP’s chemical instability under frozen storage conditions, where oxidative degradation and polymerization of catechins compromise their functional efficacy [29]. Consequently, TP typically function as a complementary antioxidant component within composite matrices rather than a standalone cryoprotectant [30], requiring stabilization through encapsulation or complexation with carriers such as cyclodextrins, chitosan, or proteins to achieve sustained activity [31].

**Figure 7:**
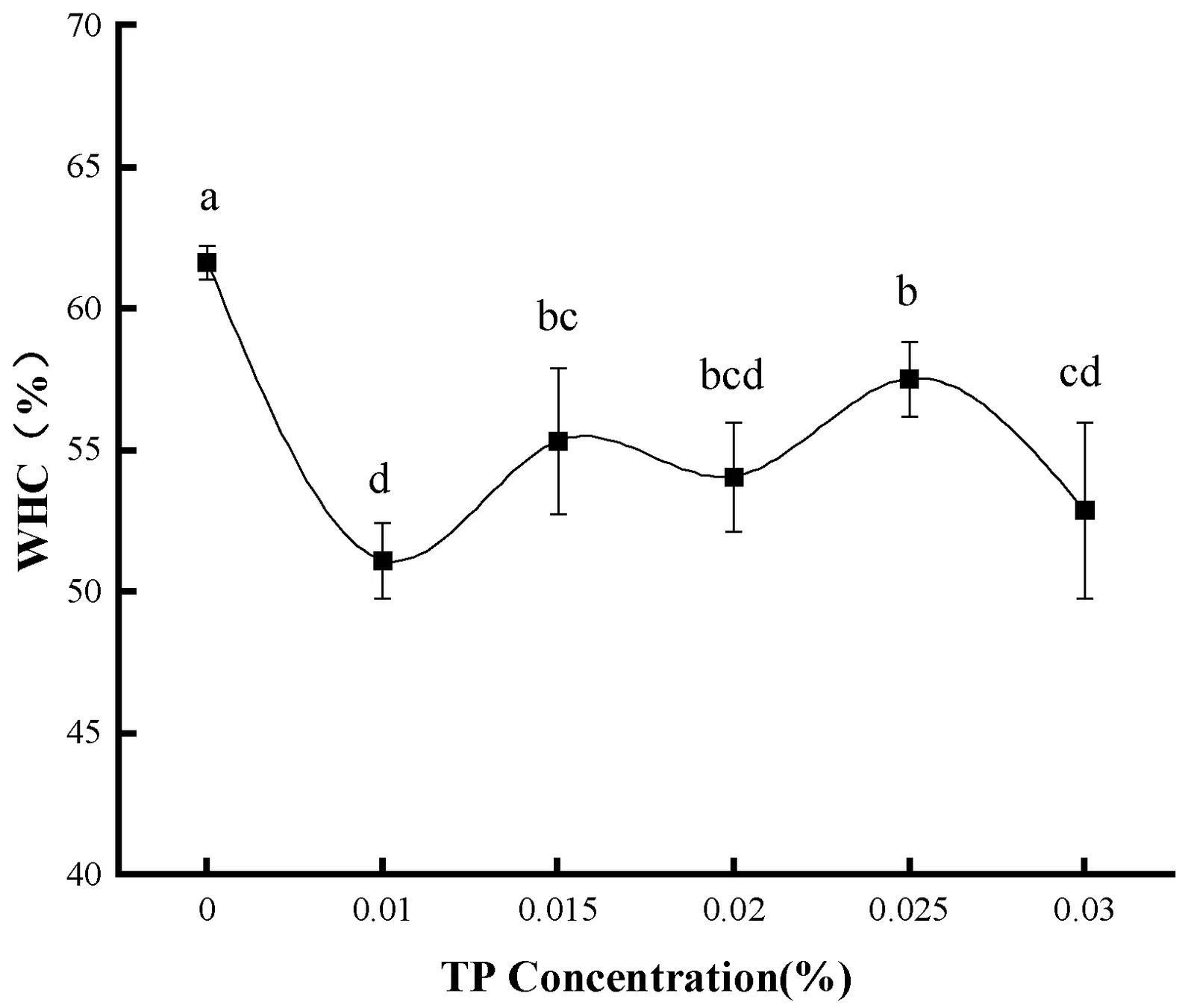
Effect of TP concentration on WHC of freshwater crayfish. Note: Data are presented as the mean ± SD (n = 3). TP refers to tea polyphenols; WHC refers to water-holding capacity. The 0% group corresponds to the untreated blank control (no cryoprotectant additives). Different lowercase letters above the bars indicate statistically significant differences (*p* < 0.05).

As illustrated in Figure 7, the blank control exhibited a WHC of 61.63 ± 0.59%. Notably, all TP-treated groups showed WHC values significantly lower than that of the blank control (*p* < 0.05), indicating that varying TP concentration within the multi-component system adversely affected moisture retention in frozen crayfish muscle. This adverse effect can be mechanistically interpreted based on the findings of Wang et al. [32]. Epigallocatechin gallate (EGCG), the primary bioactive constituent of tea polyphenols, is susceptible to oxidative conversion into quinone derivatives under frozen aqueous environments. When free EGCG accumulates locally at high concentrations, the resultant quinone moieties readily form covalent cross-links with nucleophilic amino acid residues of myosin. This triggers excessive aggregation of myofibrillar proteins and yields an overly compact gel network characterized by microscale narrow pores. Such dense microstructural architecture fails to effectively entrap immobilized water, thereby exacerbating water exudation and impairing the WHC of muscle tissue. Furthermore, unencapsulated TP undergo spontaneous oxidative polymerization during frozen storage, which drastically attenuates its radical-scavenging activity. Consequently, only trivial, non-statistically significant reductions in TBARS and TVB-N values were observed across all tested TP concentrations.

**Figure 8:**
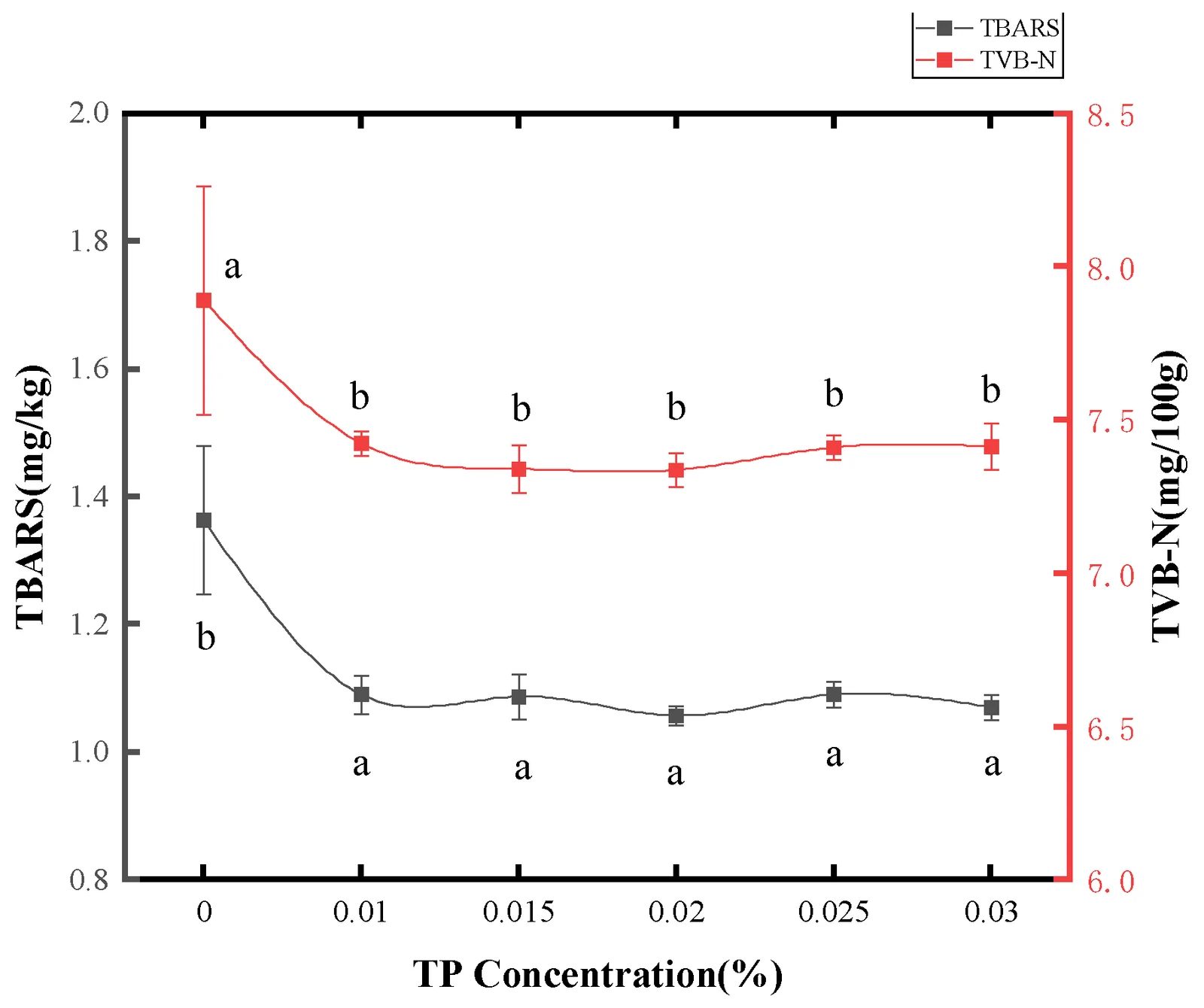
Effect of TP concentration on TVB-N and TBARS of freshwater crayfish. Note: Data are presented as the mean ± SD (n = 3). TP refers to tea polyphenols; TVB-N refers to total volatile basic nitrogen; TBARS refers to thiobarbituric acid reactive substances. The 0% group corresponds to the untreated blank control (no cryoprotectant additives). Different lowercase letters above the bars indicate statistically significant differences (*p* < 0.05).

As shown in Figure 8, the TBARS and TVB-N values of the blank control were 1.36 mg/kg and 7.89 mg/100 g, respectively, which were higher than those of all TP-treated groups. Both indicators decreased with increasing TP concentration up to 0.02%, after which they reached a plateau. This indicates that TP exhibited moderate antioxidant and bacteriostatic effects during frozen storage, consistent with their well-documented radical-scavenging activity [33].

Consequently, under the present multi-component background, adjusting TP concentration did not deliver satisfactory cryoprotective efficacy for freshwater crayfish and was excluded from subsequent response surface methodology (RSM) optimization. However, this exclusion is specific to the present unstabilized TP addition mode in this multi-component system. TP may be reintroduced in composite systems where they can be stabilized by the polysaccharide matrix or combined with emerging preservation technologies [33]. Wang et al. [33] showed that CMC-TP composite coatings effectively reduced protein oxidation and quality degradation in frozen beef, demonstrating the potential of polysaccharide–TP combinations for frozen preservation. Future studies should investigate TP incorporation in the CG-CTS-AFPs matrix in stabilized forms (e.g., β-CD encapsulated TP or chitosan–TP complexes) to leverage their antioxidant capacity without compromising the water-binding functionality of the primary cryoprotectants [34].

### 2.5. Results of Response Surface Optimization Experiment

Based on the single-factor experimental results, the concentrations of carrageenan oligosaccharides (A), chitosan (B), and AFPs (C) were selected as independent variables for a three-factor, three-level Box–Behnken design (BBD) with WHC as the response variable (Table 1). WHC showed significant negative correlations with TVB-N (*p* < 0.05) and TBARS (*p* < 0.01) (Supplementary Table S1), suggesting optimization of WHC would concurrently improve other quality attributes. Notably, TVB-N and TBARS showed modest changes between the control and optimized groups during the short-term screening period, consistent with the well-documented lag phase of lipid oxidation and protein degradation under frozen conditions, where microbial and enzymatic activities are suppressed [35]. In contrast, WHC demonstrated greater sensitivity to cryoprotectant concentration variations and directly reflects the physical efficacy of hydrogel-forming agents (CG and CTS). Moreover, WHC represents the most critical quality attribute for frozen aquatic products, directly affecting yield and consumer acceptance [36]. Therefore, WHC was selected as the most appropriate response variable for RSM optimization, while TVB-N and TBARS were monitored as secondary indicators. Subsequent validation experiments confirmed that the optimized formulation provided comprehensive quality protection across all three indicators (detailed results are presented in Section 2.6).

Using Design-Expert^®^ software, version 13.0 (Stat-Ease, Inc., Minneapolis, MN, USA), a quadratic polynomial regression model was fitted to relate WHC (Y) to the independent variables:

(1) Y = 0.7077 + 0.0022∗A + 0.0026∗B + 0.0011∗C + 0.0006∗AB + 0.0018∗AC + 0.0003∗BC − 0.0384∗A^2^ − 0.0183∗B^2^ + 0.001∗C^2^

The model demonstrated strong predictive capability, with a coefficient of determination (R^2^) of 0.9700 and an adjusted R^2^ (R^2^adj) of 0.9389, indicating an excellent fit and minimal experimental error. The complementary effect observed in the composite cryoprotectant system is attributed to the interactions between the three components. Specifically, CG and CTS are polyelectrolytes of opposite charges, which interact through electrostatic attractions and hydrogen bonding [37]. These intermolecular non-covalent associations are proposed to strengthen the molecular binding capacity for water molecules [38]. Meanwhile, AFPs embedded within this polysaccharide network provide ice crystal inhibition functionality that the polysaccharides alone cannot achieve, resulting in the observed improvement in WHC.

Analysis of variance (ANOVA) showed that the linear terms of carrageenan oligosaccharides (CG), chitosan (CTS) and AFPs were not significant (*p* > 0.05), while the quadratic terms A^2^ and B^2^ exhibited significant effects on WHC (*p* < 0.05) (Table 2). This indicates that WHC was mainly governed by quadratic nonlinear effects of CG and CTS, rather than direct linear influences from individual factors. Such a result implies that the water-holding performance may be associated with intermolecular hydrogen-bonding and non-covalent associations between polysaccharide molecules, highlighting the importance of appropriate CG and CTS concentration matching for optimal water-binding capacity. AFPs do not exert prominent linear effects on WHC, and their protective function is primarily reflected in ice-crystal regulation.

**Table 2:** ANOVA for RSM: estimated regression model of the relationship between dependent variables and independent variables.

Source	Sum of Squares	Degrees of Freedom	Mean Square	F-Value	*p*-Value
Model	0.0081	9	0.0009	58.58	<0.0001
A-CG	0.0000	1	0.0000	2.46	0.1609
B-CTS	0.0001	1	0.0001	3.61	0.0993
C-AFPs	0.0000	1	0.0000	0.6150	0.4586
AB	0.0000	1	0.0000	0.1085	0.7515
AC	0.0000	1	0.0000	0.8866	0.3778
BC	0.0000	1	0.0000	0.0192	0.8937
A^2^	0.0062	1	0.0062	404.59	<0.0001
B^2^	0.0014	1	0.0014	91.90	<0.0001
C^2^	0.0000	1	0.0000	0.2779	0.6143
Residual Error	0.0001	7	0.0000		
Lack of Fit	0.0000	3	0.0000	0.3651	0.7829
Net Error	0.0001	4	0.0000		
Total	0.0082	16			

Note: Analysis of variance (ANOVA) for the quadratic regression model fitted to WHC data. R^2^ = 0.9700; adjusted R^2^ = 0.9389. A, B, and C represent the linear terms of CG, CTS, and AFPs, respectively; A^2^, B^2^, and C^2^ are their quadratic terms; AB, AC, and BC denote interaction terms. Significant model terms are indicated by *p* < 0.05.

The model predicted the optimal cryoprotectant formulation for frozen freshwater crayfish to be 0.81% carrageenan oligosaccharides (A), 0.61% chitosan (B), and 0.17% AFPs (C), yielding a theoretical maximum WHC of 70.77%.

**Figure 9:**
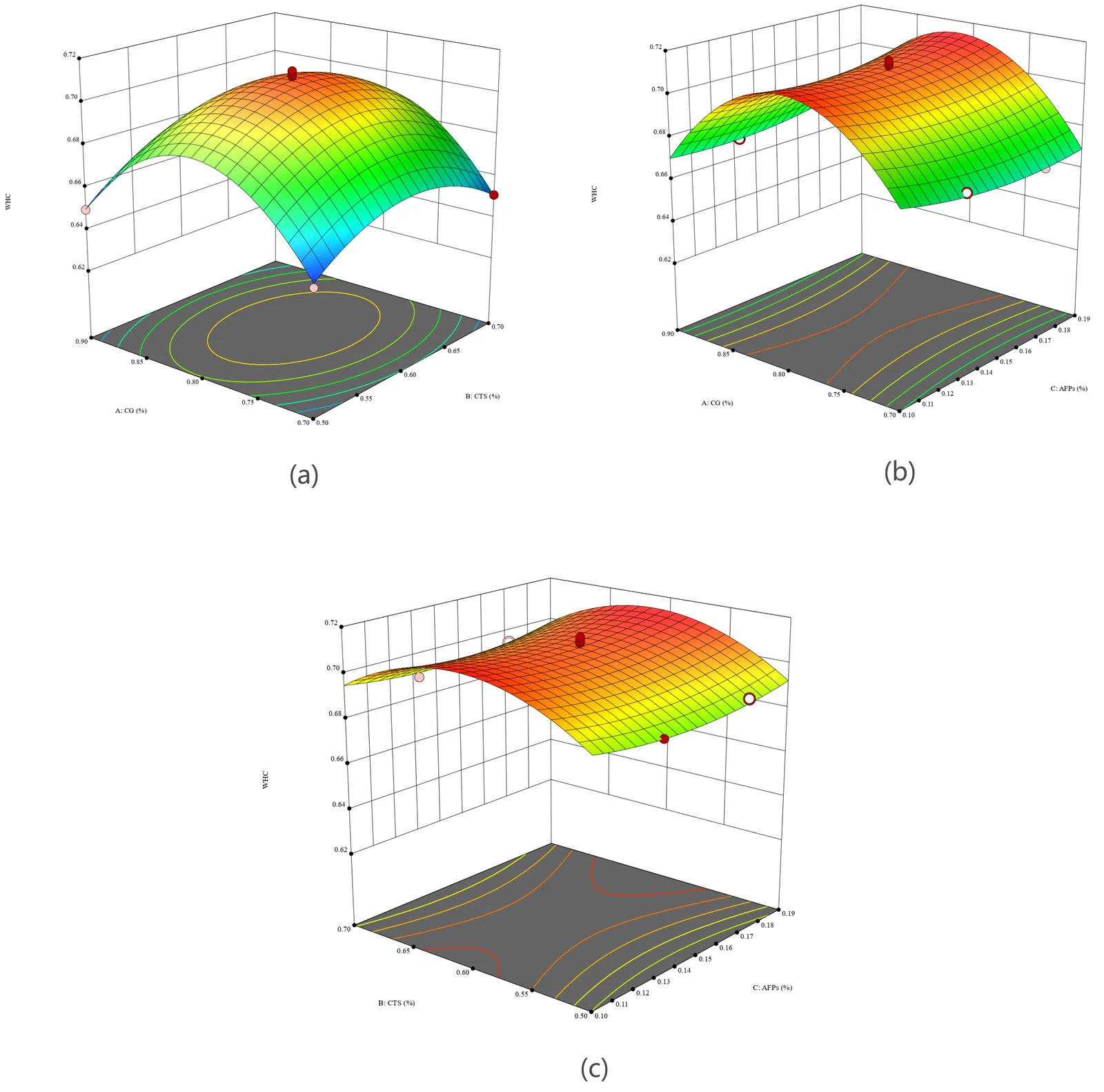
Response surface of the interactive effects of different cryoprotectants on the water holding capacity (WHC) of *Procambarus clarkii.* Note: (**a**) Surface diagram and contour diagram of the interaction between CG and CTS (**b**) Surface plot and contour plot of the interaction between CG and AFPs; (**c**) Surface plots and contour plots of the interaction between CTS and AFPs.

Response surface and contour plots (Figure 9) were generated to visualize the concentration-dependent effects and interaction strengths on WHC. The contour plots of the CG–CTS interaction show that the WHC peak occurs at moderate concentrations of both polysaccharides, suggesting that the mixed hydrogel network formed by these two components achieves optimal cross-linking density at the predicted concentrations. This optimization approach aligns with established methodologies for multicomponent formulation optimization using RSM [39], where the combination of multiple protective agents with complementary mechanisms typically outperforms single-component systems.

### 2.6. Validation of Optimal Formulation and Quality Assessment

To validate the reliability of the RSM-optimized composite cryoprotectant formulation and assess its preservation efficacy, verification experiments were conducted under the predicted optimal conditions: 0.81% carrageenan oligosaccharides (CG), 0.61% chitosan (CTS), and 0.17% antifreeze proteins (AFPs). Freshwater crayfish samples were immersed in the optimal composite solution, stored at −18 °C for 7 days, and were subsequently analyzed for quality indices. A blank control group (without cryoprotectant, stored under identical conditions) was included for comparison.

Following the 7-day frozen storage period, quality analyses revealed that the optimized group achieved a water-holding capacity (WHC) of 71.65 ± 0.46%, which closely matched the theoretical predicted value (70.77%) and was numerically higher than that of the control group (68.71 ± 0.62%). The optimized formulation exhibited significantly lower TVB-N levels compared to the control group. However, TBARS values did not differ significantly between the optimized group and the control group (*p* = 0.8318), suggesting that the current composite formulation did not exert a statistically significant inhibitory effect on lipid oxidation during this 7-day validation period (Table 3).

These findings demonstrate that the optimized composite formulation effectively maintains crayfish quality during frozen storage mainly by enhancing water-holding capacity and alleviating protein degradation. The joint mechanisms, which include intermolecular non-covalent and hydrogen-bonding interactions between CG and CTS for moisture retention, AFPs-mediated ice crystal inhibition, and the oxygen-barrier properties of polysaccharide molecular assemblies, collectively contribute to the observed cryoprotective effects. The close agreement between experimental and predicted WHC values confirms the reliability and predictive accuracy of the RSM model, supporting the practical applicability of this formulation for industrial frozen preservation of freshwater crayfish.

## 3. Conclusions

This study investigated composite cryoprotectants for low-temperature preservation of freshwater crayfish, addressing the global challenge of maintaining quality in high-moisture, high-protein aquatic products during frozen storage. Driven by consumer demand for clean-label ingredients and regulatory pressure to reduce synthetic preservatives, the development of safe and efficient composite cryoprotectant systems based on natural polysaccharide molecular assemblies represents a critical priority in sustainable food processing.

Single-factor trials combined with response surface methodology (RSM) were adopted to screen and optimize the compound formula, and the optimal compound cryoprotectant concentration was determined as 0.81% carrageenan oligosaccharides (CG), 0.61% chitosan (CTS) and 0.17% antifreeze proteins (AFPs). Verification tests showed that the water-holding capacity (WHC) of crayfish treated with the optimal formula reached 71.65 ± 0.46%, which was highly consistent with the predicted value of the regression model and markedly superior to untreated crayfish samples. In contrast, the blank control group exhibited poor overall quality after frozen storage, including lower water-holding capacity, as well as higher total volatile basic nitrogen (TVB-N) and thiobarbituric acid reactive substances (TBARS) values, confirming that crayfish muscle without cryoprotectant protection would suffer severe damage from ice crystal formation, accompanied by protein degradation and lipid oxidation. CG and CTS can suppress water migration, oxygen infiltration, and microbial reproduction through hydrogen-bonding and non-covalent intermolecular interactions, whereas AFPs mitigate ice crystal-induced tissue damage when evenly dispersed within the intermolecular interaction-formed interstitial spaces. The complementary mechanism originating from hydrogen-bonding and non-covalent interactions among mixed polysaccharides and embedded AFPs provides multi-pathway cryoprotection. The complementary action of these three substances effectively maintains the quality of frozen crayfish, offering a practical reference for the construction of polysaccharide-based composite molecular-assembly cryoprotectant systems.

In summary, this study provides a theoretical framework and practical formulation for natural polysaccharide-based composite molecular-assembly cryoprotectants (CG-CTS-AFPs) in aquatic product processing, contributing to the development of sustainable, clean-label preservation technologies while advancing the application of polysaccharide-based hydrogels in frozen food systems.

## 4. Materials and Methods

### 4.1. Raw Materials

Live freshwater crayfish (average weight of 30.0 ± 5.0 g) were purchased from RT-Mart (Chuzhou, Anhui, China) and transported to the laboratory within one hour. Chitosan (molecular weight: 150,000–300,000 Da; degree of deacetylation: 90%) and tea polyphenols (green tea extract; total polyphenols content: 98.96%; total catechins: 61.20%; EGCG: 46.67%) were purchased from Jiahe Xuri (Zhengzhou, China). Carrageenan oligosaccharides (average molecular weight ≤ 4000 Da) were obtained from Qingdao BZ Oligo Biotech Co., Ltd. (Qingdao, China). Antifreeze proteins (AFPs, purity ≥ 99%) were purchased from Shengfa Biotechnology (Liuyang, China).

### 4.2. Sample Preparation

Single-factor experiments were conducted to determine the optimal concentration range for each cryoprotectant (Table 4). Within each single-factor series, only the target cryoprotectant was varied across gradient concentrations, while the other three cryoprotectants were maintained at fixed baseline concentrations. An untreated blank control group (no cryoprotectant additives) was also prepared. This blank group was separate from the 20 experimental runs listed in Table 4. Aqueous solutions of four food-grade cryoprotectants were prepared at different mass concentrations (*w*/*v*): carrageenan oligosaccharides (0.2%, 0.4%, 0.6%, 0.8%, 1.0%), chitosan (0.3%, 0.6%, 0.9%, 1.2%, 1.5%), tea polyphenols (0.01%, 0.015%, 0.02%, 0.025%, 0.03%), and AFPs (0.05%, 0.10%, 0.15%, 0.20%, 0.25%). Specifically, the water-soluble chitosan used in this study was directly dissolved in distilled water at room temperature under continuous stirring until completely dissolved. After being anesthetized at low temperature, the crayfish were decapitated. The decapitated crayfish were immersed in their respective cryoprotectant solutions for 90 min. The samples were then tempered at 4 °C before being transferred to a −18 °C freezer (BD/BC-306JH, China Xingxing Group Co., Ltd., Taizhou, China) for final freezing. They were stored at −18 °C for 14 days before subsequent quality analysis.

### 4.3. Response Surface Experiment

Based on preliminary single-factor experiments, a three-factor, three-level Box–Behnken design (BBD) under response surface methodology (RSM) was used to investigate the effects of CG (A), CTS (B), and AFPs (C) on WHC (Y) (Table 5). The resulting data were analyzed by analysis of variance (ANOVA).

### 4.4. Determination of Water Holding Capacity (WHC)

The WHC of crayfish muscle was determined according to Fu et al. [40] with slight modifications. Crayfish muscle samples (2 g) were wrapped in filter paper and centrifuged at 6000 rpm for 10 min using a centrifuge (M16R, Shanghai Maizao Scientific Instrument Co., Ltd., Shanghai, China) at 5 °C. WHC was then calculated based on the weight loss during centrifugation and expressed as the percentage of weight retained relative to the initial sample weight.

### 4.5. Determination of Thiobarbituric Acid Reactive Substances (TBARS)

TBARS values were measured according to the method of Yang et al. with some modifications [41]. Briefly, a crayfish meat sample (0.5 g) was homogenized with 2.5 mL of a TBA solution containing 15% trichloroacetic acid, 0.375% thiobarbituric acid, and 0.25 N HCl. The mixture was heated in a boiling water bath for 10 min, cooled to room temperature under running tap water, and then centrifuged at 6000 rpm for 30 min in a centrifuge. The absorbance of the supernatant was measured at 532 nm using a UV-Vis spectrophotometer (UV-5500PC, Shanghai Yuanxi Instrument Co., Ltd., Shanghai, China). TBARS values were expressed as milligrams of malondialdehyde (MDA) per kilogram of muscle sample (mg MDA/kg).

### 4.6. Determination of Total Volatile Basic Nitrogen (TVB-N)

TVB-N content was determined using a semi-micro nitrogen method [41]. Approximately 20 g of thawed crayfish muscle was minced and homogenized with 100 mL of deionized water. The mixture was soaked for 30 min and then filtered. An aliquot (10 mL) of the filtrate was transferred to the distillation chamber of a semi-micro quantitative nitrogen analyzer, followed by the addition of 5 mL of 1% (*w*/*v*) magnesium oxide suspension (Sinopharm Chemical Reagent Co., Ltd., Shanghai, China). The distillate was collected in a receiving flask containing 10 mL of 2% (*w*/*v*) boric acid solution and five drops of a mixed indicator (0.2% methyl red and 0.1% methylene blue in ethanol). The distillate was titrated with 0.01 M hydrochloric acid solution. The TVB-N value was calculated based on the volume of acid consumed.

### 4.7. Statistical Analysis

All experiments were performed in triplicate. Data are presented as the mean ± standard deviation (SD) of three independent replicates. Statistical significance was assessed by one-way analysis of variance (ANOVA), followed by Tukey’s test for multiple comparisons, using OriginPro 2021 (OriginLab Corporation, Northampton, MA, USA). Differences were considered significant at *p* < 0.05 and highly significant at *p* < 0.01.

## Figures and Tables

**Table 1:** Experimental values of response variables for the BBD.

Std	A: CG (%)	B: CTS (%)	C: AFPs (%)	WHC (%)
1	0.70	0.50	0.15	64.49
2	0.90	0.50	0.15	64.96
3	0.70	0.70	0.15	65.10
4	0.90	0.70	0.15	65.84
5	0.70	0.60	0.13	67.07
6	0.90	0.60	0.13	66.97
7	0.70	0.60	0.17	66.72
8	0.90	0.60	0.17	67.36
9	0.80	0.50	0.13	68.71
10	0.80	0.70	0.13	68.96
11	0.80	0.50	0.17	69.07
12	0.80	0.70	0.17	69.43
13	0.80	0.60	0.15	70.54
14	0.80	0.60	0.15	70.09
15	0.80	0.60	0.15	70.97
16	0.80	0.60	0.15	71.06
17	0.80	0.60	0.15	71.22

**Table 3:** Comparison of quality attributes between control-Group and optimized composite cryoprotectant-treated *Procambarus clarkii* after frozen storage.

Group	The Control Group	The Optimized Group	*p*-Value
WHC (%)	68.71 ± 0.62	71.65 ± 0.46	0.0029
TBARS (mg/kg)	0.86 ± 0.23	0.83 ± 0.01	0.8318
TVB-N (mg/100 g)	7.71 ± 0.13	6.84 ± 0.12	0.0011

Note: Values are expressed as mean ± standard deviation (n = 3). *p*-values were obtained from an independent-sample Student’s *t*-test between the control group and the optimized group. The control group refers to crayfish stored at −18 °C for 7 days without any cryoprotectant treatment. The optimized group was treated with the predicted optimal formulation from RSM (0.81% carrageenan oligosaccharides, 0.61% chitosan, and 0.17% antifreeze protein).

**Table 4:** Single-factor experimental group design table of cryoprotectants for *Procambarus clarkii*.

Run	CG (%)	CTS (%)	AFPs (%)	TP (%)
1	0.20	0.90	0.15	0.02
2	0.40	0.90	0.15	0.02
3	0.60	0.90	0.15	0.02
4	0.80	0.90	0.15	0.02
5	1.00	0.90	0.15	0.02
6	0.60	0.30	0.15	0.02
7	0.60	0.60	0.15	0.02
8	0.60	0.90	0.15	0.02
9	0.60	1.20	0.15	0.02
10	0.60	1.50	0.15	0.02
11	0.60	0.90	0.05	0.02
12	0.60	0.90	0.10	0.02
13	0.60	0.90	0.15	0.02
14	0.60	0.90	0.20	0.02
15	0.60	0.90	0.25	0.02
16	0.60	0.90	0.15	0.010
17	0.60	0.90	0.15	0.015
18	0.60	0.90	0.15	0.020
19	0.60	0.90	0.15	0.025
20	0.60	0.90	0.15	0.030

Note: Single-factor experimental design for optimizing cryoprotectant concentrations in *Procambarus clarkii*. Each run varied only one cryoprotectant concentration while others remained fixed at baseline levels shown in the table.

**Table 5:** Factors and levels for response surface methodology.

Level	Factors		
	A (Carrageenan Oligosaccharides, %)	B (Chitosan, %)	C (Antifreeze Protein, %)
−1	0.70	0.50	0.13
0	0.80	0.60	0.15
1	0.90	0.70	0.17

Note: Coded levels (−1, 0, +1) and corresponding actual concentrations of independent variables are used in the Box–Behnken design (BBD) for response surface methodology (RSM). Coded level “0” represents the central point of the experimental domain.

## Data Availability

The original contributions presented in this study are included in the article. Further inquiries can be directed to the corresponding author.

## Supplementary Materials

The following supporting information can be downloaded at https://www.mdpi.com/article/10.3390/gels12090836/s1, Table S1: Correlation Analysis of WHC, TBARS and TVB-N in frozen *Procambarus clarkii*.

## Abbreviations

The following abbreviations are used in this manuscript:

CTS	chitosan
CG	carrageenan oligosaccharides
AFPs	antifreeze proteins
TP	tea polyphenols
WHC	water-holding capacity
TVB-N	total volatile basic nitrogen
TBARS	thiobarbituric acid reactive substances
RSM	response surface methodology
