# Supplementary material for: Effects of Hydrogel-Forming Composite Cryoprotectants on the Quality of Frozen Freshwater Crayfish (Procambarus clarkii)
Source: Gels. 2026 Sep 12;12(9):836. doi: 10.3390/gels12090836 (PMC13606180; doi:10.3390/gels12090836)
Supplement: Supplementary file 1 [file gels-12-00836-s001.zip › gels-4505450-supplementary.pdf]

Table S1 Correlation Analysis of WHC, TBARS and TVB-N in frozen *Procambarus clarkii*

Index Pairs	Pearson Correlation Coefficient	2-tailed Significance	Significance Mark	Correlation Type
TBARS vs TVB-N	0.569	0.027	*	Positive Correlation
TBARS vs WHC	-0.674	0.006	**	Negative Correlation
TVB-N vs WHC	-0.59	0.021	*	Negative Correlation

Note: * $p < 0.05$ (Significant); ** $p < 0.01$ (Highly Significant)
